# Flavonoids in *Rosa roxburghii* Tratt Fermentation Broth Ameliorate Obesity via DNMT3a/SIRT1‐Mediated Epigenetic Modulation

**DOI:** 10.1002/fsn3.70892

**Published:** 2025-09-03

**Authors:** Mi Liu, Haizhi Li, Jingzhi Zhang, Yinxue Zhong, Changyüdong Huang, Liying Zhu, Zhu Hu, Yongjie Xu, Shuyun Zhao, Wei Pan

**Affiliations:** ^1^ School of Public Health, The Key Laboratory of Environmental Pollution Monitoring and Disease Control, Ministry of Education Guizhou Medical University Guiyang China; ^2^ Prenatal Diagnosis Center The Affiliated Hospital of Guizhou Medical University Guiyang China; ^3^ Department of Obstetrics and Gynecology The Affiliated Hospital of Guizhou Medical University Guiyang China; ^4^ Department of Pathology The Affiliated Hospital of Guizhou Medical University Guiyang China; ^5^ School of Clinical Laboratory Science, Guizhou Medical University Guiyang China

**Keywords:** antioxidant, DNA methylation, obesity, *Rosa roxburghii* Tratt fermentation broth, SIRT1

## Abstract

Obesity‐related complications are often driven by chronic inflammation and oxidative stress, exacerbated by aberrant DNA methylation. Natural products with anti‐inflammatory and antioxidant properties may offer therapeutic potential. This study investigated the potential molecular mechanisms underlying the effects of *Rosa roxburghii* Tratt fermentation broth (RRTFB) on obesity through targeted methylation, while also examining its primary active components and assessing its potential therapeutic value. Male SD rats were fed a high‐fat diet (HFD) to induce obesity, with RRTFB administered as an intervention. Various methods, including reduced representation bisulfite sequencing analysis (RRBS), molecular docking, surface plasmon resonance (SPR), and other analytical methods were employed for the study. The results showed that, compared to the HFD‐fed rats, the RRTFB intervention groups (HFH and HFL) exhibited a significant reduction in MDA, IL‐6, TNF‐α and DNMT3a levels, along with increased SOD, GSH‐pX, and CAT activities in epididymal fat. RRBS revealed a significant number of differential methylation regions (DMRs) in genes related to fat metabolism, oxidative stress, and inflammation in HFD‐fed rats and HFH. Protein interaction analysis and subsequent validation experiments identified SIRT1 as a key regulator mediating the efficacy of RRTFB: RRTFB reduced SIRT1 promoter methylation and enhanced its expression. In 3T3‐L1 cells with Dnmt3a overexpression, SIRT1 levels were significantly reduced. ChIP‐qPCR further confirmed an enhanced binding of Dnmt3a to the *SIRT1* promoter. Molecular docking and SPR confirmed that flavonoids, the active components of RRTFB, could directly bind to DNMT3a and modulate its activity. This study substantiates the potential of RRTFB as a phytochemo therapeutic strategy for combating obesity, highlighting its ability to mitigate obesity through DNMT3a/SIRT1‐mediated epigenetic regulation, with flavonoids identified as the primary bioactive components.

## Graphical abstract

*Rosa roxburghii* Tratt fermentation broth alleviates obesity via DNMT3a/SIRT1 axis. RRTFB reduces fat mass, and inflammation while enhancing antioxidant activity. Flavonoids in RRTFB are the primary bioactive components.

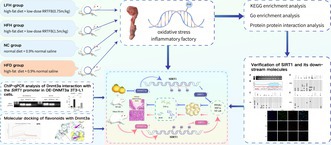

## 1 Introduction

Obesity is a significant public health concern worldwide. A report published by the Bureau of Disease Control and Prevention of the National Health Commission of China (2020) on Nutrition and Chronic Diseases of Chinese Residents indicates that about 34.3% of adult residents in China are overweight, while 16.4% are obese. It is projected that the number of overweight and obese people in China may reach approximately 790 million by 2030 (Wang et al. 2021).

Obesity is pathologically defined by the excessive and sustained accumulation of lipids in adipose tissue, which triggers dysregulated adipokines secretion. The released inflammatory factors and adipokines stimulate the occurrence of oxidative stress response (Yaribeygi et al. 2019). Critically both oxidative stress and inflammation are key drivers of obesity‐related complications (Opio et al. 2020).

Lifestyle factors exert a greater influence on obesity development than genetic predispositions. Overnutrition induces epigenetic modifications, particularly DNA methylation, which significantly contributes to obesity pathogenesis. Studies demonstrate that obese individuals display distinct global DNA methylation profiles in lymphocytes, which are associated with elevated inflammatory responses and metabolic dysregulation (Simar et al. 2014). Notably, hypermethylation near protein‐coding genes in obese subjects shows positive associations with pro‐inflammatory gene expression and a negative correlation with lipid metabolism‐related genes (Petrus et al. 2018).

Adoption of a balanced dietary pattern not only supports healthy weight maintenance but also mitigates oxidative stress and inflammatory damage. Consuming bioactive foods abundant in antioxidants represents an effective strategy for preventing and reducing oxidative stress (Pascoal et al. 2022; Galland 2010). Certain phytochemicals exhibit the ability to modulate DNA methylation, thereby potentially correcting aberrant epigenetic modification. Evidence suggests that phytochemicals can downregulate pro‐inflammatory gene expression, a process mediated through epigenetic regulation (Remely et al. 2015; Saleh et al. 2021). This provides a novel therapeutic avenue for addressing inflammatory deregulation.

*Rosa roxburghii* Tratt (RRT) is a medicinal and edible plant with significant value in traditional Chinese medicine. Research has confirmed its anti‐inflammatory (Jain et al. 2024) and antioxidant properties (Xu et al. 2020, 2021). While several studies have reported that RRT exhibits lipid‐lowering and obesity‐reducing effects, current investigations primarily focus on its modulation of intestinal microbiota (Li, Zhang, et al. 2022; Wang et al. 2022). The potential mechanism of RRT in ameliorating obesity through DNA methylation regulation remains largely unexplored.

In this study, we prepared RRT fermentation broth (RRTFB) using RRT stock solution through a 300‐days natural fermentation process without adding water or chemical additives. Fermentation enhances the synthesis of digestive enzymes and probiotics, thereby improving immune function, promoting digestion, and facilitating weight management. Previous research has demonstrated that fermented apple juice effectively regulate lipid metabolism in rats by downregulating the expression of key lipogenic genes, including fatty acid synthase, acetyl CoA carboxylase, and malate enzyme (Park et al. 2020). Therefore, this study aims to investigate the potential of RRTFB to ameliorate obesity, analyze its principal bioactive components, and elucidate the underlying mechanisms.

## 2 Materials and Methods

### 2.1 Group Grouping and Treatment of Experimental Animals

Ten‐week‐old male Sprague Dawley (SD) rats were obtained from the Animal Experiment Center of Guizhou Medical University and acclimatized for 1 week. The animal room maintains ventilation, light, a temperature of 18°C–29°C, and 40%–70% humidity, with daily provision of clean water. Rats were randomly divided into four experimental groups using a computer‐generated randomization sequence: normal control group (NC, standard diet with normal saline), obesity model group (HFD, high‐fat diet with normal saline), low‐dose RRTFB intervention group HFL, high‐fat diet with 0.75 mL/kg RRTFB daily (Xu et al. 1991)] and high‐dose RRTFB intervention group [HFH, high‐fat diet with 1.5 mL/kg RRTFB daily (Xu et al. 1991)]. RRTFB and normal saline were administered via oral gavage. After 4 weeks, two obese tolerant rats were removed, resulting in *n* = 6 per group. The success of the obese SD rats model was determined by comparing the body weight of rats fed with HFD, which was 29%–30% higher than rats fed with a standard diet. Guizhou Medical University's Experimental Animal Ethics Committee approved all animal experiments (Grant No. 2200724, 2022.03.02). RRTFB was purchased from Shanwanguo Health Industry Co. Ltd. (Lot No. 20211201, Guizhou, China). The high‐fat feed contained 59.8% ordinary feed, 20% sucrose, 18% lard, 0.2% bile salt, and 2% cholesterol. The standard feed was purchased from the Shuangshi Experimental Animal Feed Technology Co. Ltd. in Suzhou, China.

Experimental rats were treated after 24 weeks of feeding. Rats were fasted for 12 h the day before treatment. Anesthesia was induced via inhalation of isoflurane (4% for induction, 1.5%–2% for maintenance) in an oxygen carrier gas using a calibrated vaporizer (Lot No. 20220101; RWD, Shengzheng, China). Cardiac blood samples were obtained via percutaneous transthoracic. Whole blood (5–6 mL per rat) was immediately transferred to a disposable vacuum blood collection tube and centrifuged at 3000 *g* for 15 min at 4°C to isolate plasma. Tissues (liver, epididymal fat, testes) were rapidly dissected, flash‐frozen in liquid nitrogen, and stored at −80°C for subsequent analysis.

### 2.2 Biochemical Tests

Serum total cholesterol (TC, 5020‐717), triglycerides (TGs, 5010‐717), low‐density lipoprotein cholesterol (LDL‐C, 5040‐717), high‐density lipoprotein cholesterol (HDL‐C, 5030‐717), aspartate aminotransferase (AST, 1050‐717), alanine aminotransferase (ALT, 1051‐717), and glucose (GLU, Cat No: 4010‐717) were analyzed using a Beckman Coulter AU5800 automatic biochemical analyzer (California, USA). All reagents were procured from Reebio Biotechnology Ltd. (Ningbo, China).

### 2.3 Pathological Testing

Tissues were embedded in paraffin and stained with hematoxylin and eosin (HE, BA4025; BASO, Zhuhai, China). Immunohistochemical staining was conducted using the anti‐DNMT3a antibody (BS0497R; Bioss, Beijing, China). Immunofluorescence double staining was carried out on the paraffin‐embedded epididymal adipose tissue sections using antibodies against silent information regulator 1 (SIRT1) and peroxisome proliferator‐activated receptor gamma (PPARγ) antibodies (13161‐1‐AP and 16643‐1‐AP; Proteintech, Wuhan, China).

### 2.4 Enzyme‐Linked Immunosorbent Assay (ELISA)

ELISA was utilized to detect 5‐methylcytosine (5‐MC) and DNA methyltransferase (DNMT) levels (ml077383 and ml061280; Mlbio, Shanghai, China) in the liver, testis, and epididymal fat, as well as interleukin‐6 (IL‐6) and tumor necrosis factor‐alpha (TNF‐α) levels (ERC003.9 and ERC102a.9; Neobioscience, Shenzhen, China) in epididymal fat.

### 2.5 Detection of Oxidative Stress Indicators

Malondialdehyde (MDA, A003‐1‐2) and catalase (CAT, A007‐1‐1), superoxide dismutase (SOD, A001‐3‐2), and glutathione peroxidase (GSH‐Px, A005‐1‐2) activities were measured by spectrophotometry (Jiancheng Bioengineering Institute, Nanjing, China).

### 2.6 Western Blotting (WB)

Total protein samples were extracted from the epididymal adipose tissue and separated by 10% SDS‐PAGE (P1200; Solarbio, Beijing, China) and then transferred to a PVDF membrane (ISEQ00010; Merck, USA). The membranes were incubated overnight at 4°C with primary antibodies targeting TNF‐α (60291‐1‐Ig), SIRT1 (13161‐1‐AP), tumor protein P53 (TP53, 60283‐2‐Ig), hypoxia‐inducible factor 1‐alpha (HIF1a, 66730‐1‐Ig), nuclear factor‐kappa B (NF‐κB, 10745‐1‐AP) and PPARγ (16643‐1‐AP) from Proteintech (Wuhan, China), as well as IL6 (TD6087) and B‐cell lymphoma 2 (BCL2, T40056) from Abmart (Shanghai, China). After incubation with a secondary antibody, membranes were developed with an ECL substrate (P10060; NCM, Shuzhou, China) and imaged using a BioRad gel imager (CA, USA). ImageJ software (NIH, USA) was used to analyze the grayscale values and determine the expression level of the target protein. β‐Actin (ZB15001‐HRP, Servicebio, Wuhan, China) served as the reference protein.

### 2.7 DNA Methylation Sequencing and Data Analysis

DNA methylation sequencing was detected using reduced representation bisulfite sequencing (RRBS). The library was constructed and sequenced by LC Sciences (Hangzhou, China). The distribution of methylated CG, CHG, and CHH on chromosomes and gene structure was calculated for an overall assessment of methylation degree. The R package MethylKit was used to identify differentially methylated regions (DMRs), with default parameters, including 1000 bp slide windows, 500 bp overlap, and a significance threshold of *p* < 0.05. Gene Ontology (GO) and Kyoto Encyclopedia of Genes and Genomes (KEGG) pathway enrichment analyses were performed to examine DMR‐associated genes (DMGs).

### 2.8 Methylation‐Specific PCR (MSP) and Bisulfite Sequencing PCR (BSP)

Tissue genomic DNA was treated with heavy sulfite before PCR amplification. Amplification products were analyzed on a 2.0% agarose gel, and visualized with ultraviolet imaging. The tape was retrieved by cutting it, and the connection system was established. After preparing and transforming the reaction, the PCR product of the bacterial solution was generated. The bacterial solution with the correct size of the electrophoretic band was selected for sequencing. Table 1 shows the primer sequences used in this study. All reagents were obtained from Servicebio Technology Co. Ltd. (Wuhan, China).

**TABLE 1:** Used primers sequences.

Gene name	Direction	Sequences (5′ to 3′)
*SIRT1*‐BSP	Forward	TTTAGTGGGTTTAGAGAGTAGATGGG
	Reverse	CCAATTCCCACCTAAACCTCA
*SIRT1*	Forward	ATCTCCCAGATCCTCAAGCCA
	Reverse	CTTCCACTGCACAGGCACAT
*TP53*	Forward	GCTCCTCTCCCCAGCAAAAG
	Reverse	CTGGCCCTTCTTGGTCTTCG
*BCL2*	Forward	AACATCGCCCTGTGGATGAC
	Reverse	TGCACCCAGAGTGATGCAG
*PPARγ*	Forward	GGGTGAAACTCTGGGAGATTCTCC
	Reverse	CAGCAACCATTGGGTCAGCTCT
*NFκB*	Forward	ATTAGCCAGCGCATCCAGAC
	Reverse	ATCTTGAGCTCGGCAGTGTT
*HIF1a*	Forward	CACTGGACTTCGGCAGCGATGACA
	Reverse	GTGGCTTTGGAGTTTCAGAGGCAGGT
*PGC1a*	Forward	GTGCAGCCAAGACTCTGTATG
	Reverse	CGGGCTCATTGTTGTACTGGT
*DNMT3a*	Forward	ATGTGGTTCGGAGATGGCAAGTTC
	Reverse	ACCTGGAGGACTTCGTAGATGGC
*β‐Actin*	Forward	GTGCTATGTTGCTCTAGACTTCG
	Reverse	ATGCCACAGGATTCCATATACC

### 2.9 Real‐Time Quantitative PCR (RT‐qPCR)

Total RNA was extracted by Trizol Reagent (B610409; Biotechnology, Shanghai, China). The PrimeScript RT reagent kit was applied to synthesize complementary DNA (RR047A, cDNA; Takara, Otsu, Japan). RT‐qPCR was performed using the SYBR Premium Ex Taq II (DRR820A; Takara, Otsu, Japan) at Bio‐Rad CFX96 Deep Well (CA, USA). The expression level of the specific genes was determined using the 2^−∆∆CT^ method, calculated as ∆∆CT = (Experimental group cycle threshold (CT) value − Internal reference CT value) − (Control group CT value − Internal reference CT value). β‐Actin was utilized as the internal reference. The primers used in the study were synthesized by Biotech in Shanghai, China, and are listed in Table 1.

### 2.10 Construction of Cells With Overexpression of Dnmt3a Using Plasmid Vectors

Mouse embryonic fibroblasts 3T3‐L1 were cultured in a special medium from Pricella, Wuhan. The Flag‐DNMT3a expression plasmid was constructed using the pCDH‐CMV‐MCS‐EF1‐mcherry‐T2A‐Puro vector (A2523‐1; IGE, Guangzhou, China). A three‐plasmid packaging system was used to transfect HEK293T cells, and after 48 h, the virus solution was collected. 3T3‐L1 cells were treated with this solution and screened with puromycin for 72 h. Once the cells were stably transformed and growing well, total protein and RNA were extracted to confirm transfection efficiency. Transfection efficiency was verified by qPCR and western blot analysis of total RNA and protein extracts from the transduced cells.

### 2.11 Chromatin Immunoprecipitation Quantitative PCR (ChIP‐qPCR) Assay

The interaction between Dnmt3a and *SIRT1* was examined using a ChIP‐qPCR assay. 3T3‐L1 cells overexpressing Dnmt3a (OE‐DNMT3a) were cultured for 48 h before collection. The sample concentration was determined through cell crosslinking, chromatin fragmentation, DNA purification, and chromatin immunoprecipitation. RT qPCR was used for detection, with % Input calculated as: 2^(−ΔCt [normalized IP])^ × 100%. The ΔCt [normalized IP] = Ct [IP] − (Ct [Input] − Log_2_^(Input Dilution Factor)^). Primers for the *SIRT1* promoter region were synthesized by Shanghai Shenggong, with sequences: GCCATCTTCCAACTGCCTCT (Forward)/TTAAATCTCCCGCAGCCGAG (Reverse).

### 2.12 Plant Metabolomics Detection

Samples were separated using a C‐18 column (Waters, USA) and tested by an ultra‐efficient liquid chromatography system (Agilent 1290 Infinity LC, USA), along with quality control samples. The injection volume was 2 μL, the flow rate was 0.4 mL/min, and the column temperature was 40°C. An AB Triple TOF 6600 mass spectrometer was used to collect the primary and secondary spectra of samples. The secondary mass spectra were obtained by information dependent acquisition under a highly sensitive mode. Ion Source Gas1 and Gas2 were both set at 60 pa, with Curtain Gas set at 30 pa. The source temperature was maintained at 600°C, and the IonSpray Voltage was ±5500 V. Metabolite structures were determined based on the molecular weight (error < 10 ppm), retention time, collision energy, secondary fragmentation spectra, and other information of metabolites in the database, which were evaluated using the Applied Protein Technology (Shanghai, China).

### 2.13 Molecular Docking

The 3D structure of the target protein used for molecular docking analysis was obtained from the RCSB Protein Data Bank (Research Collaboratory for Structural Bioinformatics, USA; http://www.rcsb.org/). The compounds' 2D structures were derived from the PubChem database (National Institutes of Health, USA; https://pubchem.ncbi.nlm.nih.gov/). The crystal structure of the target protein was preprocessed and visualized using PyMOL software (DeLano Scientific LLC, USA), and ligand‐protein molecule docking was performed using AutoDock 4.2 software (Olson at The Scripps Research Institute, USA).

### 2.14 Surface Plasmon Resonance (SPR) Analysis

The experiment was conducted utilizing a SPR instrument (Biacore AB; GE Healthcare) and CM5 sensor chips (Cytiva, 29149603). All solutions were subjected to filtration through a 0.22 μm Millipore filter. pH 4.0 recombinant human DNMT3A protein (Full Length, GST Tag) (D353‐380G; Sino Biological, Sweden) was immobilized on the sensor chip surface through amine groups, and fisetin (HY‐N0182; MCE, USA) was injected for 1:1 binding, and the affinity between fisetin and DNMT3A was tested. All data were analyzed using Biacore Insight Evaluation Software (V 2.0.15.12933).

### 2.15 Statistical Analysis

GraphPad Prism 9 (San Diego, CA, USA) was employed for statistical analysis and plotting, and data were expressed as mean ± standard deviation (SD). Normality was assessed using the Shapiro–Wilk test, and homogeneity of variances was confirmed by Levene's test. For multiple‐group comparisons, one‐way analysis of variance (ANOVA) was performed, followed by Tukey's multiple comparisons test to identify specific inter‐group differences, and *p* < 0.05 was considered statistically significant.

## 3 Results

### 3.1 Effect of RRTFB on Mechanical and Biochemical Parameters in HFD SD Rats

**FIGURE 1:**
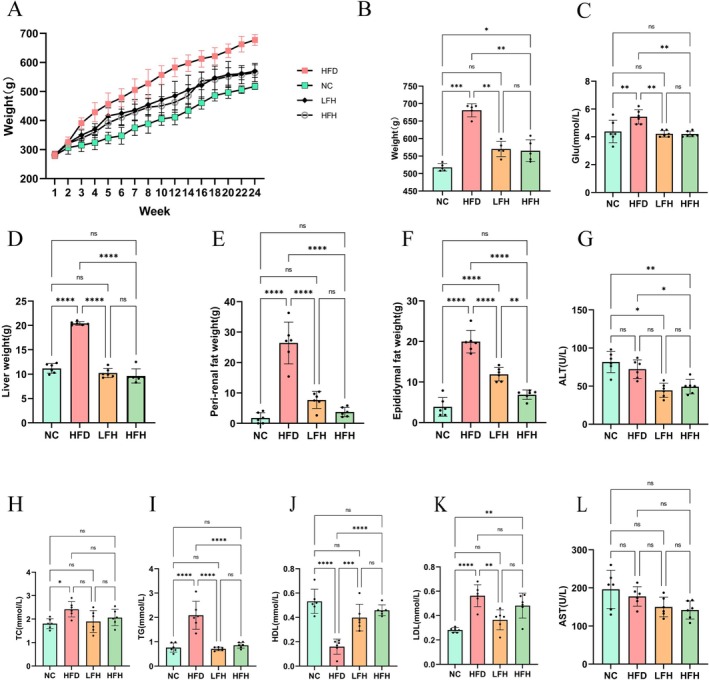
Effects of RRTFB on metabolic parameters in high‐fat diet (HFD)‐induced obese rats. (A) The weight of SD rats. (B) The weight of rats at week 24. (C) Fasting blood glucose concentration. (D) Weight of rat liver tissue. (E) Weight of perirenal fat. (F) Weight of epididymal fat. (G) Serum ALT concentration. (H) Serum TC concentration. (I) Serum TG concentration. (J) Serum HDL‐C concentration. (K) Serum LDL‐C concentration. (L) Serum AST concentration. * *p* < 0.05, ** *p* < 0.01, *** *p* < 0.001, **** *p* < 0.0001.

After 24 weeks, the HFD group rats exhibited a significantly greater body weight than those in the other groups (Figure 1A). Compared with the HFD group, the body weight of rats in the LFH and HFH groups decreased significantly (*p* < 0.05) (Figure 1B), the volume of livers that are prone to lipid deposition, perirenal adipose tissue, and epididymal adipose tissue reduced significantly (*p* < 0.05) (Figure 1D–F), the fasting blood glucose concentration decreased (*p* < 0.05) (Figure 1C), TG and LDL concentrations were significantly lower, and HDL concentrations were higher (*p* < 0.05), while TC concentration was not significantly different between the groups (Figure 1H–K). Further analysis revealed that obesity did not significantly affect liver enzymes, but it decreased ALT concentration in the HFH group compared with the HFD group (*p* < 0.05) (Figure 1G,L).

### 3.2 Effect of RRTFB on Tissue in SD Rats

**FIGURE 2:**
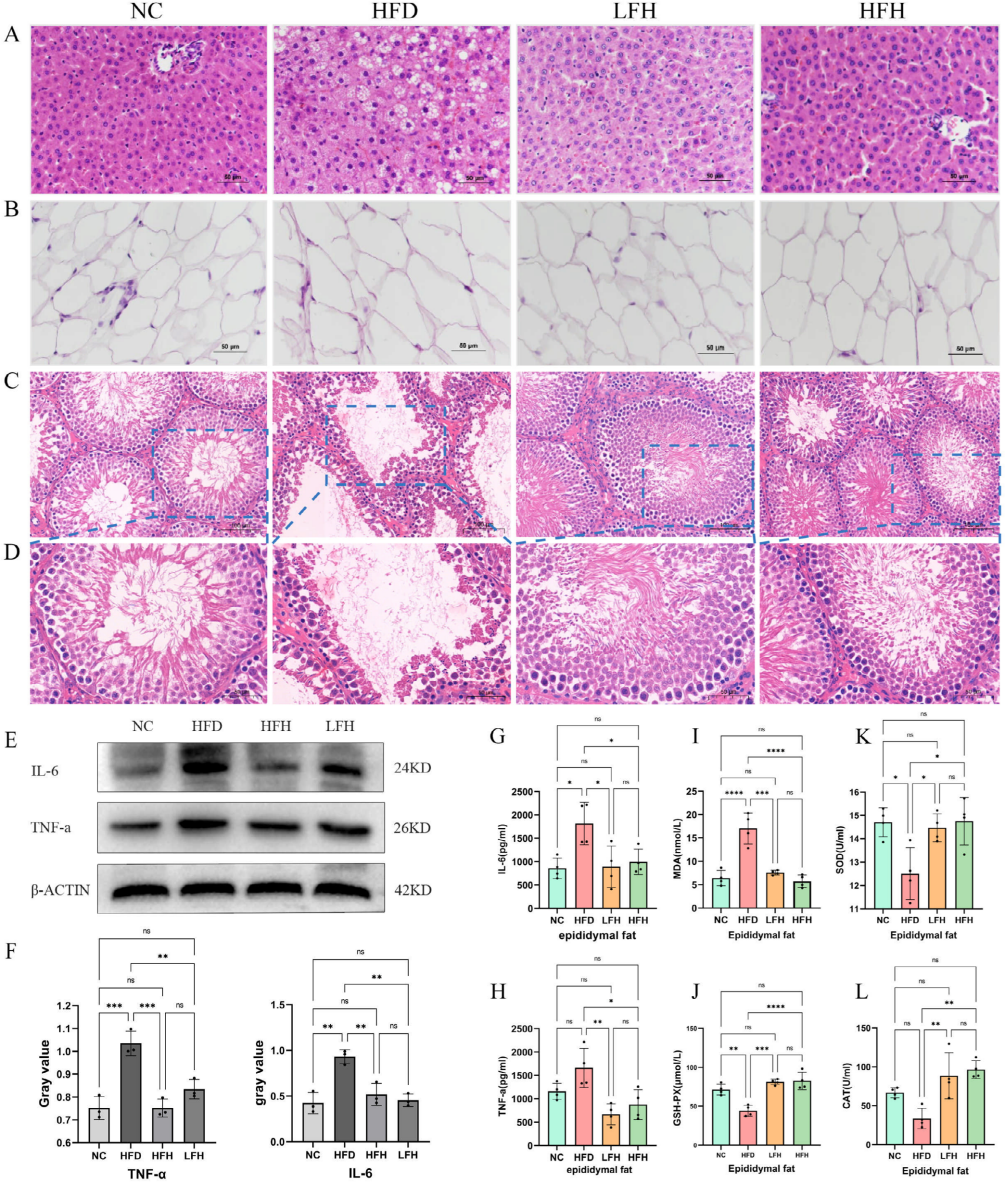
Protective effects of RRTFB against tissue damage in HFD‐induced obese rats. (A–D) HE staining results of SD rat tissues: (A) liver (200×), (B) epididymal fat (200×), (C) testis (100×), (D) testis (200×). (E–H) Changes in inflammatory cytokine levels in the epididymal fat: (E) WB results of IL‐6 and TNF‐a of epididymal fat, (F) Gray value, (G) ELISA results of IL‐6 of epididymal fat (*n* = 6), (H) ELISA results of TNF‐a of epididymal fat (*n* = 6). (I–L) Alterations in oxidative stress‐related indicators of epididymal fat (*n* = 6). * *p* < 0.05, ** *p* < 0.01, *** *p* < 0.001, **** *p* < 0.0001.

HE staining of the liver tissue showed that liver cells in the HFD group were disordered, with numerous vacuoles between cells and a pale cytoplasmic color; liver cells in the NC group were intact, with round nuclei and tight cells; liver cells in the intervention group had a significantly complete and orderly structure compared with those of the HFD group (Figure 2A). Morphological analysis of epididymal adipocytes showed that the size of adipocytes increased significantly in the HFD group, and cells decreased to varying degrees after RRTFB treatment (Figure 2B). HE staining of testicular tissue in the HFD group showed numerous empty spaces in the seminiferous tubules, disorganized sperm cell arrangement, thinner seminiferous epithelium, reduced interstitial cells, and indistinct gaps between the tubules. The shape and thickness of the seminiferous epithelium in the testes of RRTFB‐treated rats were regular, and the arrangement of seminiferous cells was relatively neat, with intact cytoplasm (Figure 2C,D).

### 3.3 Effect of RRTFB on Inflammatory Factor and Oxidative Stress in SD Rats

Compared to the HFD group, the levels of inflammatory factors (TNF‐α and IL‐6) in epididymal adipose tissue decreased significantly in the LFH and HFH groups (*p* < 0.05 for all, Figure 2E–H). Additionally, MDA levels were significantly reduced in both the LFH and HFH groups (*p* < 0.05), while there was a notable increase in the activities of SOD, CAT, and GSH‐PX (*p* < 0.05, Figure 2I–L).

### 3.4 Effect of RRTFB on Epididymal Fat Methylation in SD Rats

**FIGURE 3:**
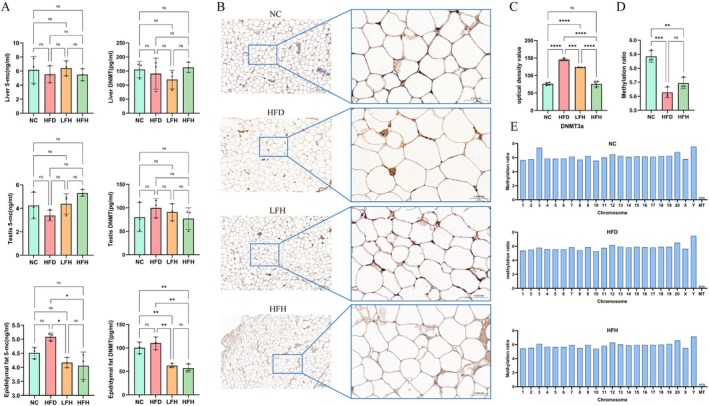
Changes in DNA methylation after intervention with RRTFB (*n* = 3). (A) ELISA detection of 5‐MC and DNMT3a levels in liver, epididymal adipose tissue, and testicular tissue. (B) Immunohistochemical analysis of DNMT3a in epididymal adipose tissue. The small image on the left is 100×, and the large image on the right is 400×. (C) Immunohistochemical average optical density value. (D) Methylation ratio by DNA methylation sequencing. (E) Methylation rate of chromosome. * * *p* < 0.01, * * * *p* < 0.001, * * * * *p* < 0.0001.

We measured 5‐MC and DNMT levels in the epididymal fat, liver, and testicular tissue of SD rats using ELISA. In epididymal fat, 5‐MC and DNMT levels decreased in the HFH and LFH groups compared to the HFD group (*p* < 0.05). No significant differences were observed in 5‐MC and DNMT levels in liver and testicular tissues between the groups (Figure 3A). Immunohistochemical analysis of DNMT3a expression in epididymal adipose tissue showed that DNMT3a levels decreased significantly in the HFH and LFH groups compared to the HFD group (*p* < 0.05) (Figure 3B).

### 3.5 DNA Methylation Sequencing

**FIGURE 4:**
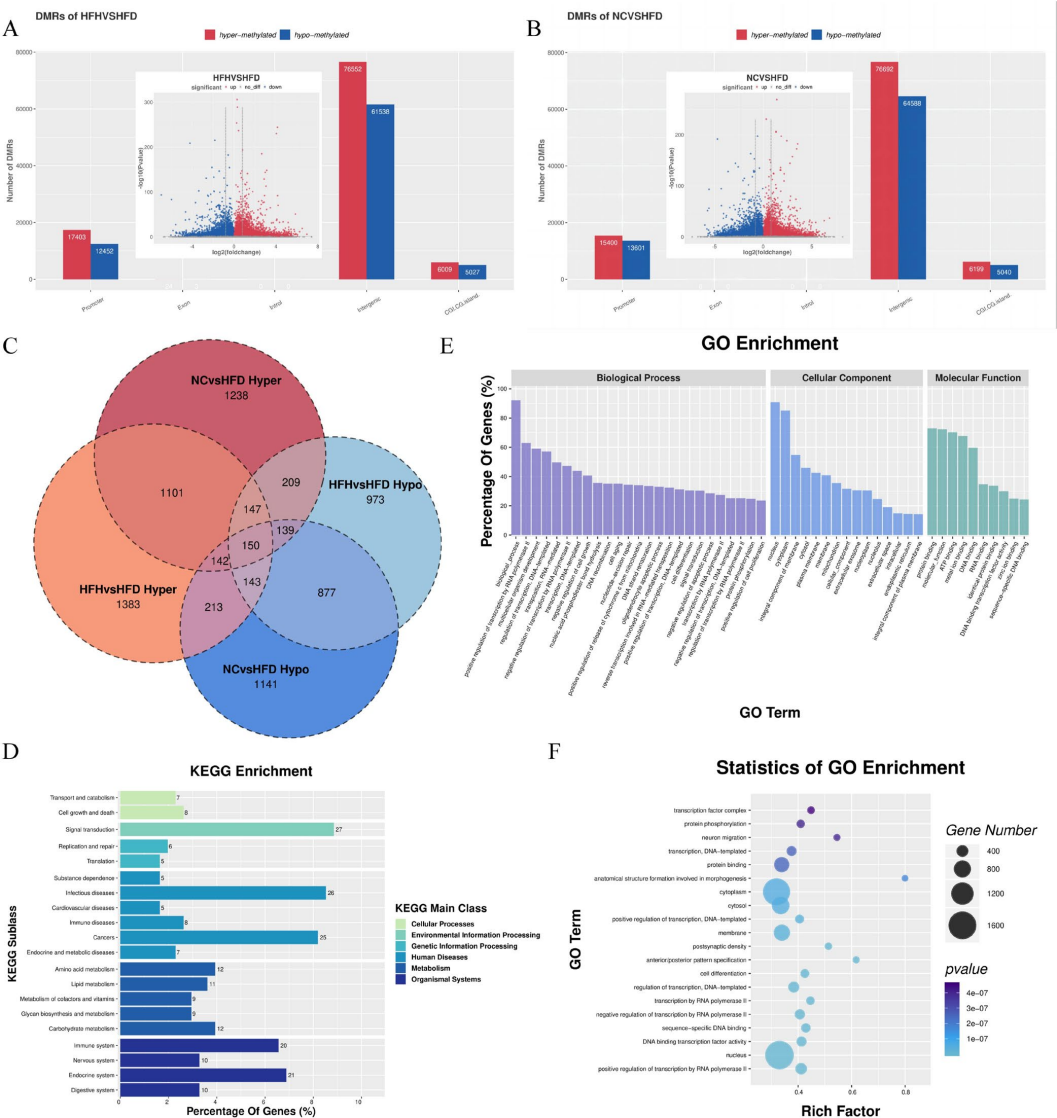
Enrichment analysis of DNA methylation sequencing results (*n* = 3). (A) DMR distribution of HFH versus HFD, with DMR volcano map in the small box. (B) DMR distribution of NC versus HFD, with DMR volcano map in the small box. (C) Venn analysis of DMG between NC versus HFD and HFH versus HFD. (D) HFH versus HFD KEGG enrichment. (E) HFH versus HFD GO enrichment. (F) HFH versus HFD GO enrichment bubble chart.

To explore the molecular mechanisms by which RRTFB alleviates obesity, particularly its impact on DNA methylation, we carried out DNA methylation sequencing on epididymal adipose tissue of male SD rats. DNA methylation sequencing revealed that the overall methylation rate in epididymal fat was higher in the NC (5.91%) and HFH (5.60%) groups compared to the HFD group (5.67%) (Figure 3D). The methylation levels of mCG (5‐methylcytosine in CpG dinucleotides), mCHG (5‐methylcytosine in CHG context), and mCHH (5‐methylcytosine in CHH context) in the HFH, HFD, and NC groups were 60.64%, 0.5%, 0.47%; 58.19%, 0.46%, 0.42%; and 60.62%, 0.47%, 0.44%, with no significant differences among the groups (Figure 3E). Using false discovery rate (FDR) < 0.05 as the selection criterion, 178,981 DMRs were identified between HFH and HFD groups, including 29,855 DMRs in promoter regions and 28,576 in gene‐associated regions, involving 6223 DMGs, among which 3432 were up‐regulated and 2791 were down‐regulated (Figure 4A). Similarly, 181,520 DMRs were identified between NC and HFD groups, including 27,852 DMRs in promoter regions and 27,737 in gene‐associated regions, involving 6223 DMGs, of which 3254 were up‐regulated and 2933 were down‐regulated (Figure 4B). Venn analysis of DMGs in the HFH and NC groups revealed 1101 up‐regulated and 877 down‐regulated genes common to both groups. This finding indicates that RRTFB intervention modified DNA methylation in epididymal adipose tissue and reversed some regions to their normal state (Figure 4C).

### 3.6 KEGG Pathway Enrichment Analyses

KEGG pathway enrichment analysis revealed that RRTFB treatment primarily affected the “Transport and catabolism” and “Cell growth and death” in terms of Cellular Processes; “Signal transduction” in terms of Environmental Information Processing; “Replication and repair” and “Translation” in terms of Genetic Information Processing; “Amino acid metabolism”, “Lipid metabolism”, “Metabolism of cofactors and vitamins”, “Glycan biosynthesis and metabolism”, and “Carbohydrate metabolism” in terms of Metabolism Processes (Figure 4D). This indicated that RRTFB can alleviate obesity symptoms by regulating intracellular metabolic pathways, signal transduction, processing of genetic information, and various metabolic processes.

### 3.7 GO Enrichment Analysis

Go enrichment analysis revealed that RRTFB treatment mainly affected the biological processes (BPs) like transcription regulation, organism development, and RNA‐mediated transposition. It also affected the cellular components such as the nucleus, cytoplasm, and membrane, as well as functions like protein binding, molecular function, and ATP binding (Figure 4E).

**FIGURE 5:**
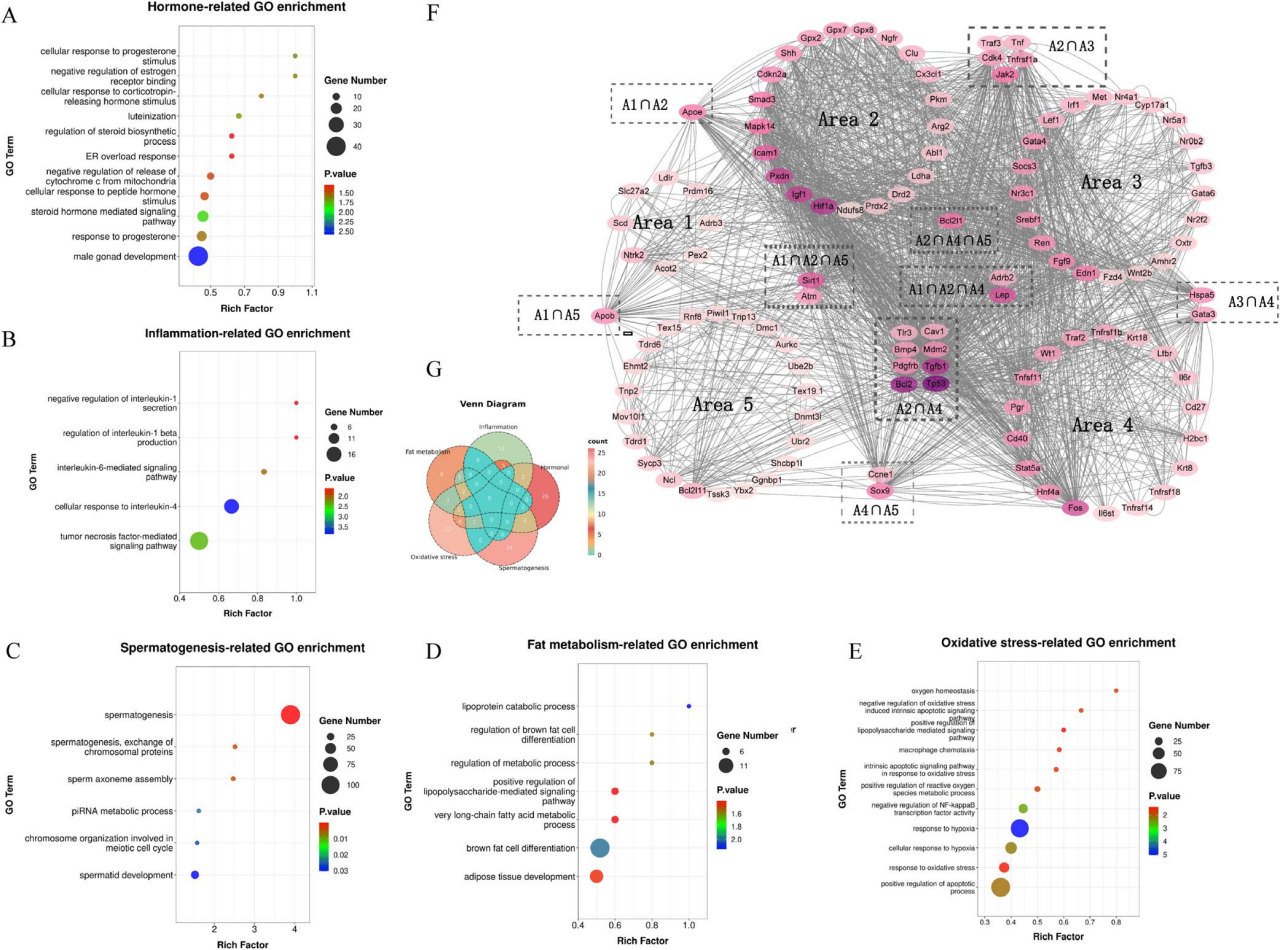
Protein interaction analysis of five main biological activities. (A) Hormone‐related GO enrichment term rich factor bubble chart. (B) Inflammation‐related GO enrichment term rich factor bubble chart. (C) Spermatogenesis‐related GO enrichment term rich factor bubble chart. (D) Fat metabolism‐related GO enrichment term rich factor bubble chart. (E) Oxidative stress‐related GO enrichment term rich factor bubble chart. (F) Five biological function‐related gene protein interaction diagrams, area 1 (A1): Fat metabolism‐related; area 2 (A2): Oxidative stress‐related; area 3 (A3): Inflammatory response‐related; area 4 (A4): Hormonal response‐related; area 5 (A5): Sperm generation‐related. The color depth of genes represents their contribution (Degree) in the entire network, with darker colors signifying a higher Degree. (G) Venn analysis of five biological function‐related genes.

Following the phenotypic changes in animal models, further studies focused specifically on GO entries related to fat metabolism, oxidative stress, inflammatory response, hormonal response, and spermatogenesis. In terms of fat metabolism, the GO enrichment entries involved include lipoprotein catabolism, very long‐chain fatty acid metabolism, adipose tissue development, etc., which involved 14 DMGs. Entries related to oxidative stress and hormonal response each involved 41 DMGs, 19 inflammatory responses, and 31 spermatogenesis. Specific BPs involved are shown in Figure 5A–E.

### 3.8 Venn and Protein Interaction Analyses

To identify key genes and proteins that interact together in different BPs, Venn and protein interaction analyses were conducted on DMRs and DMGs involved in these five types of biological activities. Venn analysis showed that SIRT1 and Ataxia‐telangiectasia mutated (ATM) are the shared genes associated with fat metabolism, oxidative stress, and sperm response. Leptin (LEP) and adrenergic receptor beta 2 (ADRB2) are the shared genes associated with fat metabolism, oxidative stress, and hormone response. The common gene in oxidative stress, hormone response, and spermatogenesis is BCL2‐like 1 (BCL2L1), a protein that regulates cell apoptosis. In the protein interaction network, TP53 had the highest Betweenness (the degree and number of centrality), followed by BCL2, transforming growth factor beta 1 (TGFB1), HIF1a, SIRT1, etc. This indicates their pivotal role in the network and may affect multiple downstream proteins and signaling pathways (Figure 5F,G).

### 3.9 Verification of Key Proteins and Detection of Downstream Molecules

Based on criteria of FDR < 0.05, a fold change (FC) exceeding 2, top 20 rankings in Degree and Betweenness, and involvement in two or more biological activities, TP53, BCL2, and SIRT1 were selected for verification (Table 2).

**TABLE 2:** Basic information on methylation of genes ranked in the top 20 by degree and betweenness.

Gene	Degree		Betweenness		Chromosome	Promoter DNA methylation region		Hyper/hypo‐methylated	*p*	FDR	FC
	Value	Rank	Value	Rank		Start	End				
*TP53*	142.00	1	2249.5	1	chr10	54299501	54300500	Hypo	0.01	0.04	0.17
					chr10	54300001	54301000	Hypo	0.00	0.00	0.32
*BCL2*	126	2	1810.9	2	chr13	22852501	22853500	Hyper	0.00	0.03	2.3
*TGFB1*	110.00	3	504.6	9	chr1	81195501	81196500	Hyper	0.00	0.04	1.82
					chr1	81196001	81197000	Hyper	0.00	0.02	1.73
					chr1	81197001	81198000	Hyper	0.00	0.00	1.28
*HIF1A*	108	4	467.9	11	chr6	92623001	92624000	Hyper	0.00	0.00	5.92
*IGF1*	102.00	5	400.6	13	chr7	22281001	22282000	Hypo	0.00	0.00	0.7
					chr7	22281501	22282500	Hypo	0.00	0.00	0.73
*PXDN*	100.00	6	356.5	17	chr6	46580001	46581000	Hypo	0.00	0.00	0.2
					chr6	46580501	46581500	Hypo	0.00	0.00	0.38
*LEP*	92	7	691.7	6	chr4	57660501	57661500	Hypo	0.00	0.02	0.81
*FOS*	92	7	358.3	16	chr6	105118001	105119000	Hypo	0.00	0.00	0.07
*SIRT1*	84.00	9	581.9	8	chr20	25306501	25307500	Hypo	0.00	0.00	0.32
					chr20	25307001	25308000	Hypo	0.00	0.01	0.44
*SOX9*	64.00	10	763.5	5	chr10	97803501	97804500	Hyper	0.00	0.00	Inf
					chr10	97804001	97805000	Hyper	0.00	0.00	Inf
					chr10	97804501	97805500	Hyper	0.00	0.00	1.9
					chr10	97805001	97806000	Hyper	0.00	0.01	1.79

**FIGURE 6:**
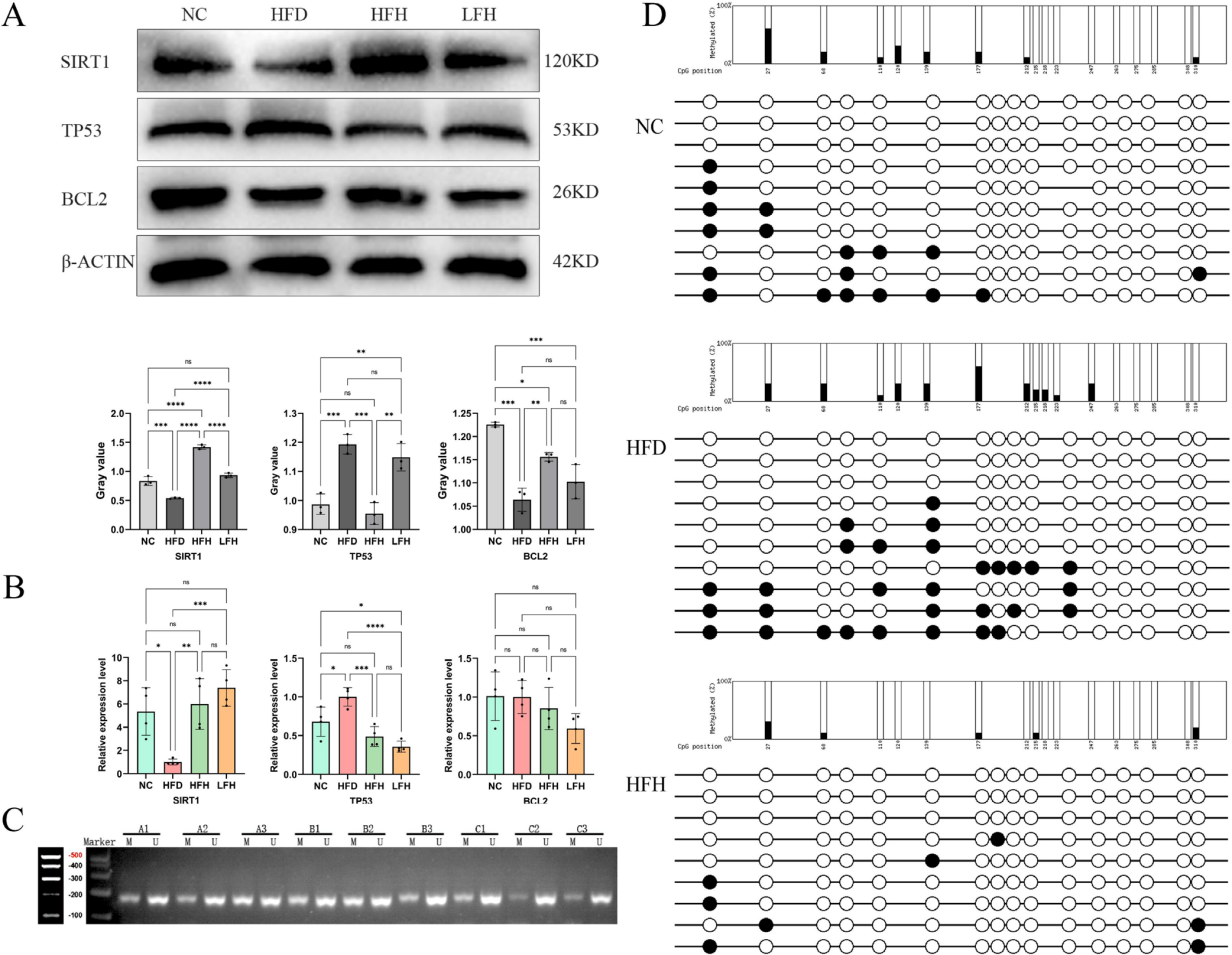
Validation of key protein and methylation changes in epididymal fat. (A) WB detect results of SIRT1, TP53, and BCL2 of epididymal fat. (B) mRNA levels of SIRT1, TP53, and BCL2 of epididymal fat. (C) MSP detection results of epididymal fat SIRT1; A1–A3: NC group, B1–B3: HFD group, C1–C3: HFH group. M, methylated; U, unmethylated. (D) BSP detection results of epididymal fat SIRT1, black color: methylated, white color: unmethylated. * *p* < 0.05, ** *p* < 0.01, *** *p* < 0.001, **** *p* < 0.0001.

RT‐qPCR and WB analysis revealed a significant decrease in TP53 and SIRT1 expression levels in both the HFH and LFH groups compared to the HFD group (*p* < 0.05). Conversely, BCL2 protein expression was significantly increased. However, no significant difference in BCL2 mRNA levels was observed between the HFH and NC groups. Furthermore, the inconsistent expression levels of TP53 and BCL2 with methylation sequencing results suggest that RRTFB may regulate these genes through mechanisms other than DNA methylation (Figure 6A,B). Fortunately, the changes in SIRT1 mRNA expression levels were consistent with the methylation sequencing results. Additionally, MSP and BSP were used to verify the methylation status of *SIRT1* in each group of rats. One CpG island(s) was found in the *SIRT1* promoter region sequence; the size is 1202 bp (start–end: 1543–2744). The results showed methylation in the *SIRT1* promoter region, with the standard deviations of percent methylated CpGs being 12.9%, 17.1%, and 8.9% in the NC, HFD, and HFH groups, respectively (Figure 6C,D). This suggests that RRTFB may significantly alleviate obesity and related chronic diseases by altering the methylation status of epididymal fat SIRT1.

### 3.10 Detection of Downstream Molecules of SIRT1

**FIGURE 7:**
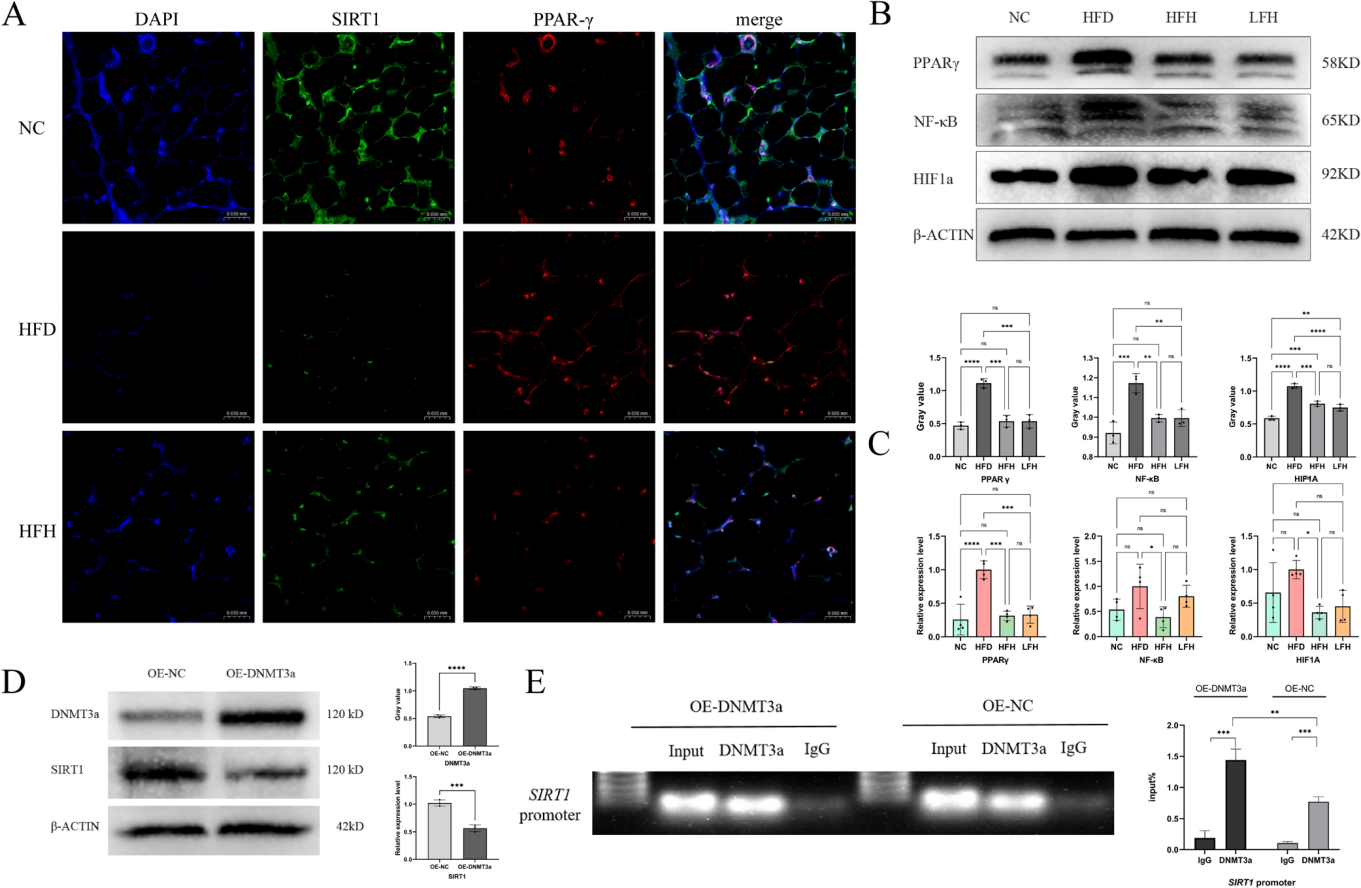
Molecular mechanisms of SIRT1 regulation in epididymal adipose tissue. (A) Immunofluorescence co‐localization of SIRT1 (green) and PPARγ (red) with nuclear DAPI staining (blue) (400×). (B) WB analysis of PPARγ, NF‐κB, and HIF1a protein expression. (C) mRNA expression levels of PPARγ, NF‐κB, and HIF1a in epididymal adipose tissue. (D) Validation of DNMT3a overexpression and its effect on SIRT1 protein levels in 3T3‐L1 adipocytes. (E) ChIP analysis of DNMT3a binding to the SIRT1 promoter region. *p* < 0.05, ** *p* < 0.01, *** *p* < 0.001, **** *p* < 0.0001.

SIRT1, a nicotinamide adenine dinucleotide (NAD)‐dependent histone deacetylase, is essential for cellular functions such as lipolysis, oxidative stress clearance, and inflammatory factors regulation. To investigate the impact of RRTFB on SIRT1 and its downstream target, PPARγ, we performed immunofluorescence double staining. In the HFD group, PPARγ expression was robust (red), while SIRT1 expression was weak (green). Following RRTFB treatment, SIRT1 expression significantly increased, whereas PPARγ expression was markedly reduced (Figure 7A). Protein and mRNA expression levels showed a consistent trend (*p* < 0.05). In addition to PPARγ, SIRT1 can also resist oxidative stress and inflammation by acetylating NF‐κB and HIF1a. Our data indicated that HIF1a and NF‐κB levels were lower in the HFH and LFH groups relative to the HFD group (Figure 7B,C).

### 3.11 The Influence of Dnmt3a Expression Levels on the Activity of SIRT1

A previous study suggested that Dnmt3a might regulate SIRT1 by methylating its promoter. To explore this, 3T3‐L1 cells overexpressing Dnmt3a (OE‐DNMT3a group) were created using gene editing. Western blot analysis showed that, compared to the control group with an empty plasmid (OE‐NC group), Dnmt3a expression was increased in the OE‐DNMT3a group, while SIRT1 levels significantly decreased (*p* < 0.001), indicating a correlation between Dnmt3a and SIRT1 (Figure 7D).

### 3.12 ChIP‐qPCR Analysis of the Interaction Between Dnmt3a and SIRT1 Promoter

To determine if Dnmt3a interacts with and binds to the *SIRT1* promoter to regulate its methylation, ChIP was performed on OE‐NC and OE‐DNMT3a groups. Results indicated Dnmt3a binds to the *SIRT1* promoter in both groups, with higher levels in OE‐DNMT3a (Figure 7E). This suggests Dnmt3a likely inhibits SIRT1 expression by promoting its hypermethylation.

### 3.13 Compound Analysis of RRTFB

**FIGURE 8:**
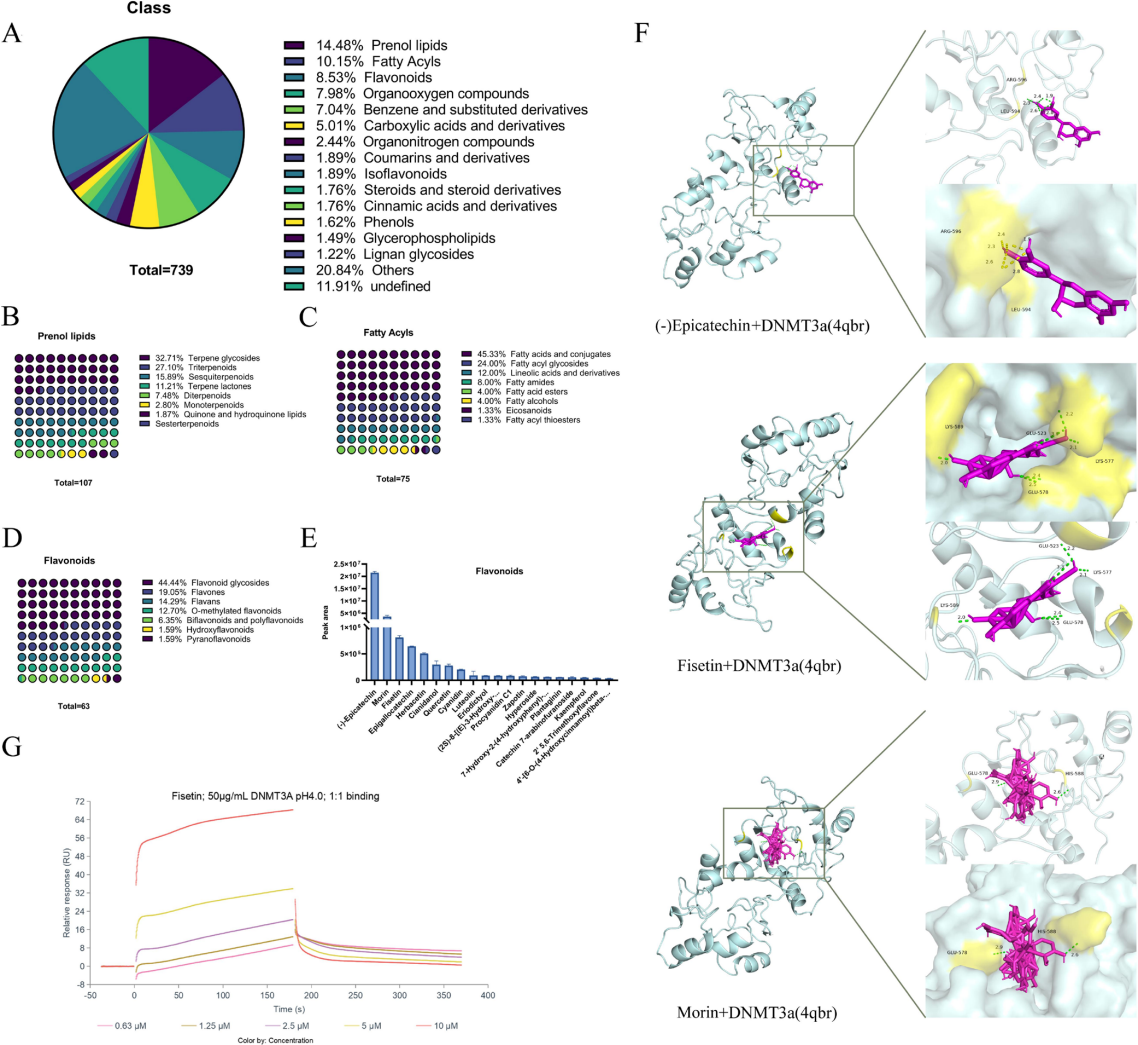
Phytochemical characterization of RRTFB and molecular docking analysis with DNMT3a. (A) Chemical classification of metabolites identified in RRTFB. (B) Subclass of prenol lipids. (C) Subclass of fatty acyls. (D) Subclass of flavonoids. (E) Relative abundance of major flavonoid components (*x*‐axis: Compounds; *y*‐axis: Normalized peak area). (F) Molecular docking models showing interactions between DNMT3a (cyan) and flavonoids (purple): (−)‐epicatechin, fisetin, morin and DNMT3a. The numerical value in the right figure represents hydrogen bonding strength, yellow represents the binding site, and the amino acids are marked in the small figure above. (G) SPR analysis of fisetin‐DNMT3a binding kinetics (*K*_d_ = 14.6 μM).

Plant metabolomics was used to analyze RRTFB's main components, utilizing both positive and negative ion patterns. Each sample was tested in triplicate. According to the classification of compounds, the top five compounds were prenol lipid (14.48%), fatty acyl (10.15%), flavonoids (8.53%), organic oxygen compounds (7.98%), benzene and its substituted derivatives (7.04%) (Figure 8A–D). Notably, (−)‐epicatechin, fisetin, and morin were identified as the three flavonoids present in the highest concentrations within RRTFB (Figure 8E).

### 3.14 Molecular Docking and SPR

To determine whether the effects of active ingredients of RRTFB were mediated by targeting DNMT3a, the top three flavonoids were molecularly docked with DNMT3a. The 3D structure 4QBR (X‐ray diffraction 1.902 Å) of DNMT3a was used for molecular docking, and the docking results revealed that the affinity of the three molecules with DNMT3a was < −6.0 kcal/mol with good activity, and the affinity from large to small was as follows: morin (−12.0 kcal/mol), fisetin (−10.6 kcal/mol), and (−)‐epicatechin (−6.8 kcal/mol) (Figure 8F). SPR analysis results revealed that the binding of fisetin to Dnmt3a was concentration‐dependent and time‐dependent, and the affinity *K*_d_ value of both was about 14.6 μm, indicating moderate binding (Figure 8G).

## 4 Discussion

Obesity and related metabolic diseases are generally believed to be associated with low‐grade chronic inflammation (Kiran et al. 2021). Adipocyte hypertrophy in obesity induces hypoxia, vascular impairment, and metabolic imbalance, triggering oxidative stress and chronic inflammation (Lee et al. 2014). Critically epigenetic dysregulation—particularly DNA methylation—may play an important role in this process (Locke et al. 2015; Cheng et al. 2018). Research has shown that obesity alters DNA methylation patterns; a study of 2515 individuals identified 94 DMRs linked to body mass index, 72 of which were linked to metabolic function (Sayols‐Baixeras et al. 2017). Notably, dietary interventions can reverse aberrant methylation status, as demonstrated by extra virgin olive oil and nuts modulating leukocyte DMRs related to metabolism, inflammation, and other factors (Arpón et al. 2017).

In this study, we reveal that RRTFB reprograms epididymal adipose methylation in HFD‐fat rats. DMRs were enriched in pathways governing fat metabolism, oxidative stress, and inflammation. Among these, SIRT1 emerged as a central regulator, consistent with its established role in adipocyte remodeling (Chen et al. 2022). Reduced SIRT1 expression in obesit (Perrini et al. 2020; Jang et al. 2017) promotes visceral adipogenesis by enhancing PPARγ activity, and can also improve inflammation and oxidative stress response by deacetylating downstream molecules such as HIF1a and NF‐κB (Shin and Lee 2017; Li, Xing, et al. 2022). Our study found that RRTFB intervention significantly reduced the levels of PPARγ, HIF1a, and NF‐κB, suggesting that RRTFB may inhibit the expression of downstream molecules related to lipid metabolism, oxidative stress, and inflammation by activating SIRT1. The downstream effects of SIRT1 activity have been extensively explored in previous studies, but the upstream regulatory mechanisms are not fully understood.

SIRT1 is modulated by multiple factors, including NAD^+^ levels, cellular energy status, and epigenetic modifications. Among these, DNA methylation serves as a critical epigenetic regulator of SIRT1 expression. Research has shown that the *SIRT1* promoter region is sensitive to methylation and can be methylated during the aging process (Erichsen and Adjaye 2022). Of note, aging and obesity exhibit significant parallels, both characterized by elevated oxidative stress, chronic inflammation, and metabolic dysfunction. Our study found that obesity induces hypermethylation of the SIRT1 gene, while RRTFB intervention reduced SIRT1 methylation and significantly upregulated its expression.

The reduction in SIRT1 methylation may be mediated by DNMT3a, a key enzyme in the DNMT family responsible for de novo methylation along with DNMT3b, which involves adding methyl to DNA regions that have not undergone methylation. DNMT3a is essential for normal DNA methylation and gene regulation, acting as a key enzyme in de novo methylation, crucial for embryonic development, cell differentiation, and gene imprinting. Our findings revealed that RRTFB treatment significantly downregulated DNMT3a expression in epididymal adipocytes, which correlated inversely with SIRT1 levels. Furthermore DNMT3a was found to bind to the *SIRT1* promoter, suggesting that the RRTFB may regulate *SIRT1* methylation by inhibiting DNMT3a activity, thereby mitigating obesity‐associated inflammation and oxidative stress.

Notably, flavonoids in RRTFB emerged as critical bioactive mediators through metabolomics analysis, molecular docking, and SPR studies that demonstrated their high‐affinity binding to DNMT3a (*K*_d_ ≈14.6 μM). These compounds exhibit well‐documented anti‐inflammatory and antioxidant properties, with emerging evidence suggesting their ability to modulate inflammatory and oxidative stress through DNA methylation regulation. Previous studies have established that curcumin can inhibit the activity of DNMT in non‐alcoholic fatty liver disease, reducing the *PPARα* promoter methylation and subsequent hepatocyte apoptosis (Li et al. 2018). Similarly, apigenin downregulates DNMT1, DNMT3a, and DNMT3b expression, leading to demethylation of the Nrf2 promoter region and enhancing the expression of this key antioxidant transcription factor (Paredes‐Gonzalez et al. 2014). These findings support our hypothesis that RRTFB‐derived flavonoids attenuate DNMT3a‐mediated *SIRT1* promoter methylation, thereby restoring its anti‐adipogenic and anti‐inflammatory functions in obesity.

While this study establishes the DNMT3a/SIRT1 axis as a mechanistic pathway for RRTFB's anti‐obesity effects, several limitations should be acknowledged. First, the exclusive use of a rodent model necessitates future validation in human adipocytes or clinical cohorts to confirm translational relevance. Second, although metabolomics identified fisetin, morin, and epicatechin as key flavonoids, their potential synergistic effects require systematic research. Third, therapeutic dosage optimization—including human‐equivalent dosing and long‐term safety assessment—remains to be established.

## 5 Conclusion

This study demonstrates that the RRTFB improves alleviates obesity and its associated metabolic complications through epigenetic regulation of the DNMT3a/SIRT1/PPARγ axis. Our findings reveal that RRTFB's bioactive flavonoids attenuate DNA hypermethylation of the *SIRT1* promoter by inhibiting DNMT3a activity, thereby restoring SIRT1‐mediated suppression of adipogenesis, inflammation and oxidative stress. While these highlights RRTFB's potential as a nutritional intervention for obesity, further investigation is required to validate these mechanisms in human systems, optimize therapeutic dosing, and characterize flavonoid synergies. Collectively, this work provides both mechanistic insights and theoretical support for the development and application of RRT.

## Author Contributions

**Mi Liu:** conceptualization (equal), data curation (equal), formal analysis (equal), funding acquisition (equal), software (equal), writing – original draft (equal). **Haizhi Li:** data curation (equal), methodology (equal), validation (equal), writing – review and editing (equal). **Jingzhi Zhang:** methodology (equal), validation (equal), writing – review and editing (equal). **Yinxue Zhong:** data curation (equal), methodology (equal). **Changyüdong Huang:** methodology (equal), software (equal). **Liying Zhu:** resources (equal). **Zhu Hu:** resources (equal). **Yongjie Xu:** conceptualization (equal), supervision (equal). **Shuyun Zhao:** conceptualization (equal), supervision (equal). **Wei Pan:** conceptualization (equal), supervision (equal).

## Ethics Statement

All animal experiments were approved by the Experimental Animal Ethics Committee of Guizhou Medical University (Grant No. 2200724, 2 March 2022).

## Conflicts of Interest

The authors declare no conflicts of interest.

## Supporting information


**Data S1:** fsn370892‐sup‐0001‐DataS1.xlsx.

## Data Availability

The original contributions presented in the study are included in the article and Supporting Information. Further inquiries can be directed to the corresponding author.
